# Epiglottic retroversion as a cause of upper airway obstruction: A case report

**DOI:** 10.1097/MD.0000000000037142

**Published:** 2024-02-09

**Authors:** Seung Jun Lee, Manbong Heo, Jong Hwan Jeong, Ji-Ho Park, Chang Min Lee, Seong Jun Won, Jong Deog Lee

**Affiliations:** a Division of Pulmonology and Allergy, Department of Internal Medicine, Gyeongsang National University Hospital, Gyeongsang National University School of Medicine, Jinju, South Korea; b Department of Surgery, Gyeongsang National University Hospital, Gyeongsang National University School of Medicine, Jinju, South Korea; c Division of Gastroenterology, Department of Internal Medicine, Gyeongsang National University Hospital, Gyeongsang National University School of Medicine, Jinju, South Korea; d Department of Otolaryngology, Gyeongsang National University Hospital, Gyeongsang National University School of Medicine, Jinju, South Korea.

**Keywords:** airway obstruction, epiglottis, respiratory failure, retroversion

## Abstract

**Rationale:**

Epiglottic retroversion is the abnormal movement of the epiglottis to the rima glottis, resulting in blockage of inspiratory airflow. Acute upper airway obstruction caused by epiglottic retroversion can lead to sudden respiratory failure. Epiglottic retroversion has occasionally been reported in horses and dogs; however it is extremely rare in humans. Herein, we report a case of epiglottic retroversion causing recurrent upper airway obstruction in human.

**Patient concerns:**

We present the case of a 74-year-old man who was diagnosed with epiglottic retroversion without evidence of epiglottis. The patient presented with recurrent episodes of abnormal breathing sounds and dyspnea. Inspiratory stridor was evident whenever the patient experienced dyspnea.

**Diagnosis:**

Epiglottic retroversion was diagnosed as the cause of upper airway obstruction using fiber-optic bronchoscopy.

**Interventions:**

The patient underwent tracheostomy to prevent acute respiratory failure because the recurrent episodes of stridor and dyspnea did not improve.

**Outcomes:**

The episodic dyspnea and oxygen desaturation did not relapse after tracheostomy and he could be discharged home.

**Lessons:**

This case highlights the importance of considering epiglottic retroversion as a cause of acute upper airway obstruction.

## 1. Introduction

Upper airway obstruction is a life-threatening condition that may lead to acute respiratory failure and sudden death. The supraglottic larynx, including the arytenoids, aryepiglottic folds, epiglottis, and ventricular fold, is the most common site for upper airway obstruction.^[1]^ The etiology of upper airway obstruction varies widely. Congenital diseases are common in children, and malignant neoplasms, such as glottic and subglottic cancers, are common in adults. In addition, several nonmalignant diseases cause upper airway obstruction in adults. Common causes include laryngomalacia and infectious conditions such as epiglottitis, supraglottitis, and neck abscess.^[2]^

The roles and functions of the epiglottis during breathing are mostly unknown. Amis et al reported that the human epiglottis remains nearly stationary during ordinary breathing.^[3]^ Thus, acute upper airway obstruction caused by abnormal epiglottic movements is extremely rare in humans. Herein, we report a case of epiglottic retroversion that caused recurrent upper airway obstruction without accompanying infection. The patient and the legal guardian provided informed consent for publication of this case report.

## 2. Case report

A 74-year-old man, who was bedridden for over 5 years due to Parkinson disease, presented with sudden onset of abnormal breathing sounds and dyspnea. He was a former smoker with a smoking history of 40 pack-years, and his medical history included angina. A history of recurrent hospital admission for the treatment of aspiration pneumonia during the previous 2 years was noted. The patient had visited the hospital 14 days earlier due to acute abdominal pain in the right lower quadrant, and laparoscopic appendectomy was performed under general anesthesia for the treatment of acute appendicitis. His abdominal pain subsided, and his general condition markedly improved after surgery. However, the patient’s caregiver complained that he suddenly made an abnormal sound during respiration, and dyspnea accompanied by noisy breathing sounds was observed. Cough and purulent sputum were absent, and the patient did not have fever.

On physical examination, the patient appeared chronically ill and dyspneic. His vital signs were as follows: blood pressure, 125/75 mm Hg; body temperature, 36.6 °C; heart rate, 110 beats per minute; and oxygen saturation, 97% on 2 L/min oxygen via a nasal prong. The respiratory rate was 28 breaths per minute, and inspiratory stridor was audible in the supine position.

**Figure 1.**
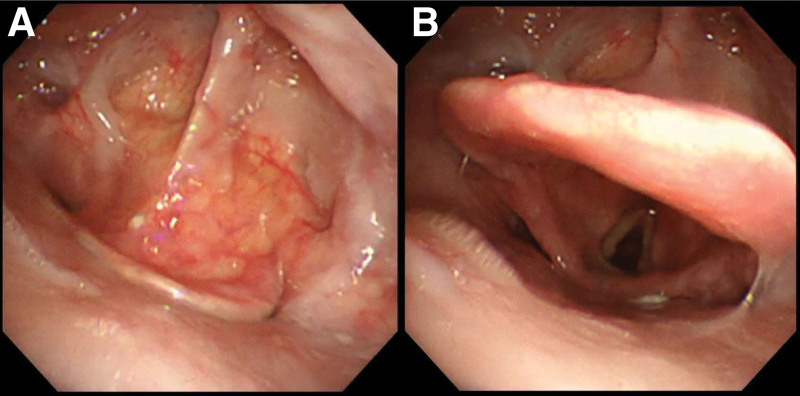
Bronchoscopy images during inspiration (A) and expiration (B) show epiglottic retroflexion causing airflow blockage during inspiration.

Initial laboratory tests showed a white blood cell count of 5480/mm^3^ with 80.1% neutrophils and 17.9% lymphocytes, hemoglobin level of 11.9 g/dL, platelet count of 222,000/mm^3^, C-reactive protein level of 26.9 mg/L, and erythrocyte sedimentation rate of 98 mm/h. No definite abnormalities were observed on chest radiography. Arterial blood gas analysis revealed a pH of 7.47, PaCO_2_ of 38 mm Hg, PaO_2_ of 76 mm Hg, and oxygen saturation of 96.0% on room air. The patient experienced recurrent episodes of oxygen desaturation and dyspnea during hospitalization. Inspiratory stridor accompanied every episode of desaturation and dyspnea. Interestingly, inspiratory stridor was present in the supine and sitting positions, but was absent when the patient was lying on the lateral side. Fiber-optic bronchoscopy was performed to determine the cause of suspected upper airway narrowing. The apex of the epiglottis excessively moved downward during inspiration and blocked the inspiratory airflow to the laryngeal airway (Fig. 1 and Video S1, Supplemental Digital Content, http://links.lww.com/MD/L328; video of bronchoscopy in the beginning and patient’s video in the latter part). Abnormal retroflexion of the epiglottis, called epiglottic retroversion, was confirmed as the cause of inspiratory stridor and oxygen desaturation in this patient. There was no evidence of epiglottitis or vocal cord dysfunction. We consulted to otolaryngologist for the treatment of epiglottic retroversion. Partial resection of epiglottis or tracheostomy could be considered as a treatment strategy. After careful discussion we concluded that surgical resection could not guarantee the reversal of upper airway obstruction and risk of complication related to the epiglottic resection was relatively high. A tracheostomy was finally performed to prevent acute respiratory failure due to upper airway obstruction. The episodic dyspnea and oxygen desaturation did not relapse after tracheostomy and he could be discharged home.

## 3. Discussion

A case series including 5 patients with asthma-mimicking inappropriate laryngeal closure was published in 1983.^[4]^ This report suggested that laryngeal obstruction can occur without any structural or neurological problems. Thirty years later, the term inducible laryngeal obstruction (ILO) was defined and accepted at the European Respiratory Society/European Laryngological Society/American College Chest Physicians International Consensus Conference.^[5]^ The term ILO is now widely used to describe laryngeal obstruction causing breathing problems.^[6]^

Exercise is a common cause of ILO, and in exercise-induced laryngeal obstruction (EILO), abnormalities of the larynx are induced by increased ventilatory demands during exercise.^[7]^ The typical presenting symptoms are dyspnea and inspiratory stridor in proportion to the exercise intensity. Fretheim-Kelly et al suggested that epiglottic retroversion is a specific type of EILO in equines.^[7]^ Epiglottic retroversion is the retroflexion of the epiglottic apex and the coverage of the rima glottis by the epiglottis, which causes obstruction of the laryngeal entrance and limits the airflow in the upper airway.^[7]^ Epiglottic retroversion is extremely rare in humans; thus, limited data exist. Walsted et al reported laryngoscopic findings during exercise in patients with EILO from 5 international expert centers.^[8]^ Closure at the arytenoid level was the most common finding, and vocal-cord dysfunction was seen in 18% of patients.^[8]^ The authors did not comment on epiglottic retroversion, although unclassifiable subtypes were reported in 3% of patients.^[8]^ Upper airway obstruction caused by epiglottic retroversion has been reported in 2 patients.^[9,10]^ Both patients had a history of head trauma. Traumatic injury of the hypoglossal nerve and dysfunction of the pharyngeal muscles may have caused of epiglottic retroversion in these cases. The cause of epiglottic retroversion in the present case was unclear because the patient did not have a history of head trauma or evidence of epiglottitis. Thus, this is very important limitation of the present case. We can postulate that general anesthesia, Parkinson disease, and bedridden state are possible precipitating factors for epiglottic retroversion in this patient. However, functional test to investigate dysfunction of hypoglossal nerve or phryngeal muscle could not be performed. This case highlights the importance of considering epiglottic retroversion as a cause of acute upper airway obstruction.

## Author contributions

**Conceptualization:** Seung Jun Lee, Jong Deog Lee.

**Investigation:** Seong Jun Won.

**Methodology:** Manbong Heo Heo, Jong Hwan Jeong.

**Supervision:** Jong Deog Lee.

**Resources:** Ji-Ho Park, Chang Min Lee.

**Visualization:** Seung Jun Lee, Seong Jun Won.

**Writing – original draft:** Seung Jun Lee.

**Writing – review & editing:** Manbong Heo Heo, Jong Hwan Jeong, Jong Deog Lee.

## Supplementary Material


